# 4,4′-[Piperazine-1,4-diylbis(propyl­ene­nitrilo­methyl­idyne)]diphenol

**DOI:** 10.1107/S1600536809045620

**Published:** 2009-11-04

**Authors:** Ruibo Xu, Xingyou Xu, Xujie Yang, Yuping Huang, Chunmei Xu

**Affiliations:** a Department of Chemical Engineering, Huaihai Institute of Technology, Lianyungang 222005, People’s Republic of China; b Huaiyin Institute of Technology, Huaian 223003, People’s Republic of China; c Materials Chemistry Laboratory, Nanjing University of Science & Technology, Nanjing 210094, People’s Republic of China

## Abstract

In the title mol­ecule, C_24_H_32_N_4_O_2_, the piperazine ring adopts a chair conformation and the dihedral angle between the two benzene rings is 35.4 (1)°. In the crystal structure, inter­molecular O—H⋯N hydrogen bonds link mol­ecules into chains along [001].

## Related literature

For the properties of piperazine derivatives, see: Keypour *et al.* (2008 ▶, 2009 ▶); Paital *et al.* (2009 ▶). For related structures, see: Thirumurugan *et al.* (1998 ▶); Yogavel *et al.* (2003 ▶).
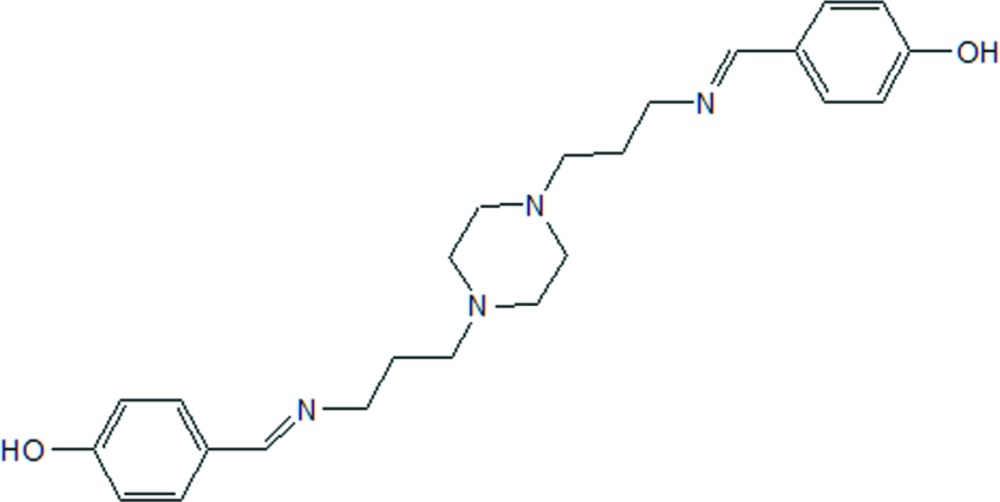


## Experimental

### 

#### Crystal data

C_24_H_32_N_4_O_2_
*M* *_r_* = 408.54
Monoclinic, 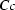

*a* = 5.9701 (10) Å
*b* = 30.159 (3) Å
*c* = 12.8348 (18) Å
β = 97.558 (2)°
*V* = 2290.9 (6) Å^3^
*Z* = 4
Mo *K*α radiation
μ = 0.08 mm^−1^
*T* = 298 K
0.17 × 0.15 × 0.11 mm

#### Data collection

Siemens SMART CCD diffractometer
Absorption correction: multi-scan (*SADABS*; Sheldrick, 1996 ▶) *T*_min_ = 0.987, *T*_max_ = 0.992
5997 measured reflections
2016 independent reflections
1387 reflections with *I* > 2σ(*I*)
*R*_int_ = 0.068

#### Refinement

*R*[*F* ^2^ > 2σ(*F* ^2^)] = 0.043
*wR*(*F* ^2^) = 0.103
*S* = 0.96
2016 reflections
271 parameters
2 restraints
H-atom parameters constrained
Δρ_max_ = 0.14 e Å^−3^
Δρ_min_ = −0.19 e Å^−3^

### 

Data collection: *SMART* (Siemens, 1996 ▶); cell refinement: *SAINT* (Siemens, 1996 ▶); data reduction: *SAINT*; program(s) used to solve structure: *SHELXS97* (Sheldrick, 2008 ▶); program(s) used to refine structure: *SHELXL97* (Sheldrick, 2008 ▶); molecular graphics: *SHELXTL* (Sheldrick, 2008 ▶); software used to prepare material for publication: *SHELXTL*.

## Supplementary Material

Crystal structure: contains datablocks I, global. DOI: 10.1107/S1600536809045620/lh2937sup1.cif

Structure factors: contains datablocks I. DOI: 10.1107/S1600536809045620/lh2937Isup2.hkl

Additional supplementary materials:  crystallographic information; 3D view; checkCIF report

## Figures and Tables

**Table 1:** Hydrogen-bond geometry (Å, °)

*D*—H⋯*A*	*D*—H	H⋯*A*	*D*⋯*A*	*D*—H⋯*A*
O2—H2⋯N1^i^	0.82	1.98	2.762 (4)	159
O1—H1⋯N2^ii^	0.82	2.00	2.780 (4)	159

Symmetry codes: (i) 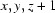; (ii) 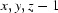.

**Fig. 1.**
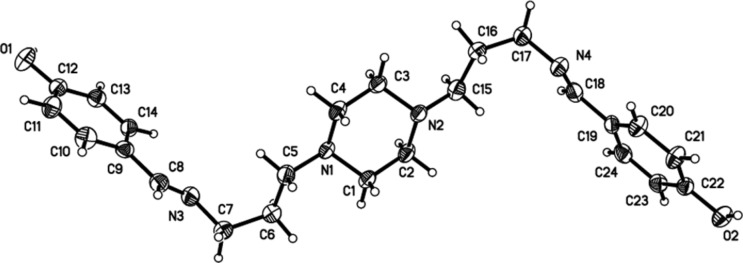
The molecular structure of (1). Displacement ellipsoids are drawn at the 30% probability level.

**Fig. 2.**
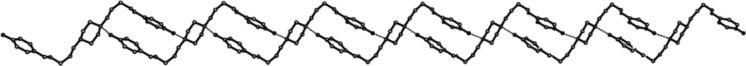
One-dimensional chain structure of (1) constructed by O—H···N intermolecular hydrogen bonds (dashed lines) along [001].

## supplementary crystallographic information

### Comment

Piperazine derivatives possesses interesting structures and properties (Yogavel *et al.*, 2003; Thirumurugan *et al.*, 1998; Keypour *et al.*, 2008,2009; Paital *et al.*, 2009), therefore, our group have designed and prepared series of Schiff bases and complexes derived from substituted piperazines. As part of our work, the title compound (I) a potential hexadentate Schiff base ligand, was synthesized in our group and herein we report the crystal structure.

The molecular structure of (I) is shown in Fig. 1. In (I), the C8—N3 and C18—N4 double bond lengths are comparable to reported values (Yogavel *et al.*, 2003; Thirumurugan *et al.*, 1998). The dihedral angle between the two benzene rings (C9—C14 and C19—C24) is 35.4 (1) °. In the piperazine ring, atoms C1/C2/C3/C4 are essentially planar (the mean deviation from the plane is 0.0032 Å), and atoms N1 and N2 atom lie 0.6936 and 0.6693 Å either side of this plane. This four atom plane makes a dihedral angle of 50.9 (3)° with the plane of atoms C2/N2/C3 and 52.7 (3) ° with atoms C1/N1/C4, respectively, in accordance with the chair conformation of the piperazine ring. In the crystal structure, intermolecular O—H···N hydrogen bonds link molecules into one-dimensional chains along [001](Fig.2).

### Experimental

A solution of *N*,*N*'-bis(*N*-aminopropyl)-piperazine (1.5 mmol in 10 ml anhydrous methanol) was added dropwise with constant stirring to a solution of parahydroxybenzaldehyde (3 mmol in 15 ml anhydrous methanol) at 325 K for 3 h. The resulting mixture was filtered. After cooling, the filtrate was evaporated at ambient environment. Several days later, pink crystals suitable for X-ray analysis were collected and washed with small amount of methanol and dried at room temperature.

### Refinement

H atoms were placed in calculated positions with C—H = 0.97 Å(piperazinyl), 0.93 Å(benzene), 0.82 Å(hydroxyl) and 0.97 Å(methylene), and refined in riding mode with *U*_iso_(H)= 1.5 *U*_eq_(O) and *U*_iso_(H) = 1.2*U*_eq_(C).

### Crystal data

**Table:**

C_24_H_32_N_4_O_2_	*F*(000) = 880
*M**_r_* = 408.54	*D*_x_ = 1.185 Mg m^−^^3^
Monoclinic, *C**c*	Mo *K*α radiation, λ = 0.71073 Å
Hall symbol: C -2yc	Cell parameters from 1760 reflections
*a* = 5.9701 (10) Å	θ = 2.7–24.9°
*b* = 30.159 (3) Å	µ = 0.08 mm^−^^1^
*c* = 12.8348 (18) Å	*T* = 298 K
β = 97.558 (2)°	Lamellate, pink
*V* = 2290.9 (6) Å^3^	0.17 × 0.15 × 0.11 mm
*Z* = 4	

### Data collection

**Table:**

Siemens SMART CCD diffractometer	2016 independent reflections
Radiation source: fine-focus sealed tube	1387 reflections with *I* > 2σ(*I*)
graphite	*R*_int_ = 0.068
φ and ω scans	θ_max_ = 25.0°, θ_min_ = 1.4°
Absorption correction: multi-scan (*SADABS*; Sheldrick, 1996)	*h* = −7→6
*T*_min_ = 0.987, *T*_max_ = 0.992	*k* = −30→35
5997 measured reflections	*l* = −15→15

### Refinement

**Table:**

Refinement on *F*^2^	Primary atom site location: structure-invariant direct methods
Least-squares matrix: full	Secondary atom site location: difference Fourier map
*R*[*F*^2^ > 2σ(*F*^2^)] = 0.043	Hydrogen site location: inferred from neighbouring sites
*wR*(*F*^2^) = 0.103	H-atom parameters constrained
*S* = 0.96	*w* = 1/[σ^2^(*F*_o_^2^) + (0.0507*P*)^2^] where *P* = (*F*_o_^2^ + 2*F*_c_^2^)/3
2016 reflections	(Δ/σ)_max_ < 0.001
271 parameters	Δρ_max_ = 0.14 e Å^−^^3^
2 restraints	Δρ_min_ = −0.19 e Å^−^^3^

### Special details

**Table:**

Geometry. All e.s.d.'s (except the e.s.d. in the dihedral angle between two l.s. planes) are estimated using the full covariance matrix. The cell e.s.d.'s are taken into account individually in the estimation of e.s.d.'s in distances, angles and torsion angles; correlations between e.s.d.'s in cell parameters are only used when they are defined by crystal symmetry. An approximate (isotropic) treatment of cell e.s.d.'s is used for estimating e.s.d.'s involving l.s. planes.
Refinement. Refinement of *F*^2^ against ALL reflections. The weighted *R*-factor *wR* and goodness of fit *S* are based on *F*^2^, conventional *R*-factors *R* are based on *F*, with *F* set to zero for negative *F*^2^. The threshold expression of *F*^2^ > σ(*F*^2^) is used only for calculating *R*-factors(gt) *etc*. and is not relevant to the choice of reflections for refinement. *R*-factors based on *F*^2^ are statistically about twice as large as those based on *F*, and *R*- factors based on ALL data will be even larger.

### Fractional atomic coordinates and isotropic or equivalent isotropic displacement parameters (Å^2^)

**Table:**

	*x*	*y*	*z*	*U*_iso_*/*U*_eq_	
N1	0.6528 (5)	0.39796 (9)	0.5395 (2)	0.0440 (7)	
N2	0.6327 (5)	0.34403 (9)	0.7270 (2)	0.0450 (7)	
N3	0.6917 (7)	0.47133 (10)	0.2563 (2)	0.0619 (9)	
N4	0.5753 (7)	0.26995 (10)	1.0120 (2)	0.0604 (9)	
O1	1.0094 (5)	0.38009 (9)	−0.1521 (2)	0.0684 (8)	
H1	0.8981	0.3653	−0.1744	0.103*	
O2	0.2701 (4)	0.37289 (8)	1.40788 (19)	0.0592 (7)	
H2	0.3880	0.3735	1.4486	0.089*	
C1	0.5611 (7)	0.41527 (12)	0.6330 (3)	0.0509 (10)	
H1A	0.4439	0.4369	0.6113	0.061*	
H1B	0.6804	0.4300	0.6789	0.061*	
C2	0.4643 (7)	0.37801 (12)	0.6919 (3)	0.0524 (10)	
H2A	0.4043	0.3901	0.7526	0.063*	
H2B	0.3403	0.3644	0.6467	0.063*	
C3	0.7284 (7)	0.32708 (12)	0.6338 (3)	0.0510 (10)	
H3A	0.6111	0.3122	0.5871	0.061*	
H3B	0.8464	0.3057	0.6560	0.061*	
C4	0.8247 (6)	0.36472 (12)	0.5762 (3)	0.0504 (10)	
H4A	0.9453	0.3788	0.6226	0.060*	
H4B	0.8890	0.3530	0.5163	0.060*	
C5	0.7518 (7)	0.43362 (13)	0.4807 (3)	0.0552 (10)	
H5A	0.8202	0.4203	0.4237	0.066*	
H5B	0.8712	0.4479	0.5273	0.066*	
C6	0.5862 (8)	0.46839 (13)	0.4356 (3)	0.0628 (11)	
H6A	0.5490	0.4873	0.4920	0.075*	
H6B	0.4481	0.4540	0.4044	0.075*	
C7	0.6779 (9)	0.49699 (13)	0.3524 (3)	0.0680 (12)	
H7A	0.5796	0.5224	0.3361	0.082*	
H7B	0.8268	0.5079	0.3798	0.082*	
C8	0.8797 (8)	0.46994 (12)	0.2204 (3)	0.0555 (11)	
H8	1.0022	0.4850	0.2563	0.067*	
C9	0.9117 (7)	0.44553 (11)	0.1246 (3)	0.0479 (9)	
C10	1.1116 (7)	0.44905 (13)	0.0817 (3)	0.0608 (10)	
H10	1.2280	0.4664	0.1153	0.073*	
C11	1.1427 (7)	0.42709 (14)	−0.0112 (3)	0.0584 (10)	
H11	1.2774	0.4304	−0.0395	0.070*	
C12	0.9734 (6)	0.40044 (12)	−0.0611 (3)	0.0471 (9)	
C13	0.7736 (6)	0.39566 (12)	−0.0171 (3)	0.0501 (10)	
H13	0.6592	0.3775	−0.0497	0.060*	
C14	0.7435 (6)	0.41775 (12)	0.0751 (3)	0.0472 (9)	
H14	0.6100	0.4140	0.1041	0.057*	
C15	0.5266 (7)	0.30844 (12)	0.7830 (3)	0.0566 (10)	
H15A	0.4159	0.2933	0.7332	0.068*	
H15B	0.4465	0.3219	0.8360	0.068*	
C16	0.6907 (8)	0.27448 (12)	0.8355 (3)	0.0591 (11)	
H16A	0.8220	0.2896	0.8713	0.071*	
H16B	0.7411	0.2555	0.7821	0.071*	
C17	0.5864 (9)	0.24597 (14)	0.9141 (3)	0.0703 (13)	
H17A	0.4353	0.2372	0.8841	0.084*	
H17B	0.6759	0.2193	0.9286	0.084*	
C18	0.3869 (7)	0.28242 (12)	1.0347 (3)	0.0530 (10)	
H18	0.2584	0.2760	0.9879	0.064*	
C19	0.3598 (7)	0.30661 (11)	1.1315 (3)	0.0446 (9)	
C20	0.5407 (7)	0.31283 (13)	1.2093 (3)	0.0563 (11)	
H20	0.6820	0.3019	1.1994	0.068*	
C21	0.5156 (7)	0.33506 (13)	1.3016 (3)	0.0566 (10)	
H21	0.6392	0.3387	1.3530	0.068*	
C22	0.3063 (6)	0.35184 (11)	1.3173 (3)	0.0462 (9)	
C23	0.1256 (7)	0.34603 (12)	1.2404 (3)	0.0517 (9)	
H23	−0.0150	0.3574	1.2501	0.062*	
C24	0.1506 (7)	0.32352 (12)	1.1488 (3)	0.0545 (10)	
H24	0.0260	0.3196	1.0980	0.065*	

### Atomic displacement parameters (Å^2^)

**Table:**

	*U*^11^	*U*^22^	*U*^33^	*U*^12^	*U*^13^	*U*^23^
N1	0.0460 (18)	0.0507 (17)	0.0371 (16)	0.0038 (15)	0.0126 (15)	−0.0067 (14)
N2	0.0486 (18)	0.0493 (17)	0.0378 (16)	0.0054 (15)	0.0086 (14)	−0.0041 (14)
N3	0.086 (3)	0.053 (2)	0.047 (2)	−0.0058 (19)	0.0136 (19)	−0.0030 (17)
N4	0.084 (3)	0.0542 (19)	0.045 (2)	0.0001 (19)	0.0140 (19)	−0.0078 (16)
O1	0.0580 (18)	0.092 (2)	0.0577 (17)	−0.0007 (15)	0.0161 (15)	−0.0221 (16)
O2	0.0517 (17)	0.0707 (18)	0.0567 (17)	0.0012 (14)	0.0131 (14)	−0.0143 (14)
C1	0.060 (2)	0.054 (2)	0.041 (2)	0.0106 (19)	0.0137 (19)	−0.0060 (18)
C2	0.049 (2)	0.067 (2)	0.043 (2)	0.012 (2)	0.0145 (19)	−0.0047 (19)
C3	0.057 (2)	0.054 (2)	0.043 (2)	0.0123 (19)	0.0086 (19)	−0.0054 (18)
C4	0.045 (2)	0.065 (3)	0.042 (2)	0.0102 (18)	0.0110 (18)	−0.0056 (19)
C5	0.063 (3)	0.058 (2)	0.046 (2)	−0.007 (2)	0.014 (2)	−0.0035 (19)
C6	0.083 (3)	0.052 (2)	0.056 (3)	0.003 (2)	0.019 (2)	−0.002 (2)
C7	0.105 (4)	0.049 (2)	0.052 (2)	−0.004 (2)	0.019 (2)	−0.008 (2)
C8	0.070 (3)	0.049 (2)	0.047 (2)	−0.005 (2)	0.004 (2)	−0.0016 (18)
C9	0.055 (2)	0.043 (2)	0.044 (2)	0.0047 (18)	0.0010 (19)	0.0022 (17)
C10	0.054 (3)	0.069 (3)	0.057 (2)	−0.009 (2)	−0.001 (2)	−0.007 (2)
C11	0.046 (2)	0.073 (3)	0.056 (2)	0.001 (2)	0.008 (2)	−0.003 (2)
C12	0.047 (2)	0.051 (2)	0.042 (2)	0.0076 (18)	0.0023 (19)	0.0014 (19)
C13	0.052 (2)	0.049 (2)	0.049 (2)	−0.0023 (18)	0.003 (2)	−0.0043 (18)
C14	0.049 (2)	0.049 (2)	0.045 (2)	0.0014 (18)	0.0081 (18)	0.0016 (18)
C15	0.059 (2)	0.064 (2)	0.048 (2)	−0.008 (2)	0.0096 (19)	−0.005 (2)
C16	0.083 (3)	0.050 (2)	0.045 (2)	0.012 (2)	0.012 (2)	−0.0052 (19)
C17	0.113 (4)	0.052 (2)	0.048 (2)	0.001 (3)	0.018 (3)	−0.005 (2)
C18	0.066 (3)	0.049 (2)	0.042 (2)	−0.011 (2)	0.001 (2)	0.0043 (18)
C19	0.055 (2)	0.045 (2)	0.0339 (19)	−0.0059 (18)	0.0061 (17)	0.0028 (16)
C20	0.052 (2)	0.068 (3)	0.051 (2)	0.000 (2)	0.014 (2)	−0.010 (2)
C21	0.047 (2)	0.077 (3)	0.046 (2)	−0.006 (2)	0.005 (2)	−0.016 (2)
C22	0.050 (2)	0.046 (2)	0.044 (2)	−0.0031 (19)	0.010 (2)	−0.0013 (18)
C23	0.047 (2)	0.055 (2)	0.053 (2)	0.0088 (19)	0.006 (2)	0.0049 (19)
C24	0.054 (2)	0.058 (2)	0.047 (2)	0.001 (2)	−0.0086 (19)	0.0080 (19)

### Geometric parameters (Å, °)

**Table:**

N1—C4	1.467 (5)	C8—C9	1.466 (5)
N1—C1	1.478 (4)	C8—H8	0.9300
N1—C5	1.481 (4)	C9—C10	1.383 (5)
N2—C2	1.465 (5)	C9—C14	1.395 (5)
N2—C15	1.479 (4)	C10—C11	1.398 (5)
N2—C3	1.483 (4)	C10—H10	0.9300
N3—C8	1.269 (5)	C11—C12	1.381 (6)
N3—C7	1.467 (5)	C11—H11	0.9300
N4—C18	1.256 (5)	C12—C13	1.392 (5)
N4—C17	1.458 (5)	C13—C14	1.390 (5)
O1—C12	1.361 (4)	C13—H13	0.9300
O1—H1	0.8200	C14—H14	0.9300
O2—C22	1.367 (4)	C15—C16	1.513 (6)
O2—H2	0.8200	C15—H15A	0.9700
C1—C2	1.511 (5)	C15—H15B	0.9700
C1—H1A	0.9700	C16—C17	1.520 (5)
C1—H1B	0.9700	C16—H16A	0.9700
C2—H2A	0.9700	C16—H16B	0.9700
C2—H2B	0.9700	C17—H17A	0.9700
C3—C4	1.509 (5)	C17—H17B	0.9700
C3—H3A	0.9700	C18—C19	1.468 (5)
C3—H3B	0.9700	C18—H18	0.9300
C4—H4A	0.9700	C19—C20	1.384 (5)
C4—H4B	0.9700	C19—C24	1.394 (5)
C5—C6	1.504 (6)	C20—C21	1.386 (5)
C5—H5A	0.9700	C20—H20	0.9300
C5—H5B	0.9700	C21—C22	1.387 (5)
C6—C7	1.529 (6)	C21—H21	0.9300
C6—H6A	0.9700	C22—C23	1.374 (5)
C6—H6B	0.9700	C23—C24	1.382 (5)
C7—H7A	0.9700	C23—H23	0.9300
C7—H7B	0.9700	C24—H24	0.9300
			
C4—N1—C1	107.4 (3)	C10—C9—C8	120.7 (4)
C4—N1—C5	110.5 (3)	C14—C9—C8	121.2 (4)
C1—N1—C5	111.9 (3)	C9—C10—C11	121.5 (4)
C2—N2—C15	109.7 (3)	C9—C10—H10	119.3
C2—N2—C3	108.3 (3)	C11—C10—H10	119.3
C15—N2—C3	112.1 (3)	C12—C11—C10	120.0 (4)
C8—N3—C7	118.2 (4)	C12—C11—H11	120.0
C18—N4—C17	119.5 (4)	C10—C11—H11	120.0
C12—O1—H1	109.5	O1—C12—C11	118.1 (3)
C22—O2—H2	109.5	O1—C12—C13	122.8 (3)
N1—C1—C2	110.5 (3)	C11—C12—C13	119.1 (3)
N1—C1—H1A	109.5	C14—C13—C12	120.6 (3)
C2—C1—H1A	109.5	C14—C13—H13	119.7
N1—C1—H1B	109.5	C12—C13—H13	119.7
C2—C1—H1B	109.5	C13—C14—C9	120.7 (3)
H1A—C1—H1B	108.1	C13—C14—H14	119.7
N2—C2—C1	112.5 (3)	C9—C14—H14	119.7
N2—C2—H2A	109.1	N2—C15—C16	114.4 (3)
C1—C2—H2A	109.1	N2—C15—H15A	108.7
N2—C2—H2B	109.1	C16—C15—H15A	108.7
C1—C2—H2B	109.1	N2—C15—H15B	108.7
H2A—C2—H2B	107.8	C16—C15—H15B	108.7
N2—C3—C4	110.4 (3)	H15A—C15—H15B	107.6
N2—C3—H3A	109.6	C15—C16—C17	112.4 (4)
C4—C3—H3A	109.6	C15—C16—H16A	109.1
N2—C3—H3B	109.6	C17—C16—H16A	109.1
C4—C3—H3B	109.6	C15—C16—H16B	109.1
H3A—C3—H3B	108.1	C17—C16—H16B	109.1
N1—C4—C3	112.1 (3)	H16A—C16—H16B	107.9
N1—C4—H4A	109.2	N4—C17—C16	111.1 (3)
C3—C4—H4A	109.2	N4—C17—H17A	109.4
N1—C4—H4B	109.2	C16—C17—H17A	109.4
C3—C4—H4B	109.2	N4—C17—H17B	109.4
H4A—C4—H4B	107.9	C16—C17—H17B	109.4
N1—C5—C6	114.6 (3)	H17A—C17—H17B	108.0
N1—C5—H5A	108.6	N4—C18—C19	123.2 (4)
C6—C5—H5A	108.6	N4—C18—H18	118.4
N1—C5—H5B	108.6	C19—C18—H18	118.4
C6—C5—H5B	108.6	C20—C19—C24	117.8 (3)
H5A—C5—H5B	107.6	C20—C19—C18	121.0 (4)
C5—C6—C7	112.6 (4)	C24—C19—C18	121.2 (4)
C5—C6—H6A	109.1	C19—C20—C21	121.3 (4)
C7—C6—H6A	109.1	C19—C20—H20	119.3
C5—C6—H6B	109.1	C21—C20—H20	119.3
C7—C6—H6B	109.1	C20—C21—C22	120.1 (4)
H6A—C6—H6B	107.8	C20—C21—H21	120.0
N3—C7—C6	110.8 (3)	C22—C21—H21	120.0
N3—C7—H7A	109.5	O2—C22—C23	118.2 (3)
C6—C7—H7A	109.5	O2—C22—C21	122.6 (3)
N3—C7—H7B	109.5	C23—C22—C21	119.1 (3)
C6—C7—H7B	109.5	C22—C23—C24	120.7 (3)
H7A—C7—H7B	108.1	C22—C23—H23	119.6
N3—C8—C9	122.7 (4)	C24—C23—H23	119.6
N3—C8—H8	118.6	C23—C24—C19	120.9 (4)
C9—C8—H8	118.6	C23—C24—H24	119.5
C10—C9—C14	118.1 (3)	C19—C24—H24	119.5
			
C4—N1—C1—C2	−57.8 (4)	O1—C12—C13—C14	−178.9 (3)
C5—N1—C1—C2	−179.3 (3)	C11—C12—C13—C14	0.7 (6)
C15—N2—C2—C1	−179.4 (3)	C12—C13—C14—C9	0.8 (5)
C3—N2—C2—C1	−56.8 (4)	C10—C9—C14—C13	−2.5 (5)
N1—C1—C2—N2	59.2 (4)	C8—C9—C14—C13	178.4 (3)
C2—N2—C3—C4	56.1 (4)	C2—N2—C15—C16	−172.3 (3)
C15—N2—C3—C4	177.3 (3)	C3—N2—C15—C16	67.3 (4)
C1—N1—C4—C3	59.4 (4)	N2—C15—C16—C17	165.2 (3)
C5—N1—C4—C3	−178.3 (3)	C18—N4—C17—C16	109.1 (5)
N2—C3—C4—N1	−60.0 (4)	C15—C16—C17—N4	−77.3 (5)
C4—N1—C5—C6	177.5 (3)	C17—N4—C18—C19	179.8 (3)
C1—N1—C5—C6	−62.9 (4)	N4—C18—C19—C20	−7.1 (5)
N1—C5—C6—C7	−164.6 (3)	N4—C18—C19—C24	173.7 (3)
C8—N3—C7—C6	−125.9 (4)	C24—C19—C20—C21	0.1 (5)
C5—C6—C7—N3	70.6 (5)	C18—C19—C20—C21	−179.0 (3)
C7—N3—C8—C9	−179.6 (3)	C19—C20—C21—C22	−0.4 (6)
N3—C8—C9—C10	172.5 (4)	C20—C21—C22—O2	178.0 (3)
N3—C8—C9—C14	−8.3 (5)	C20—C21—C22—C23	0.1 (5)
C14—C9—C10—C11	2.7 (6)	O2—C22—C23—C24	−177.6 (3)
C8—C9—C10—C11	−178.1 (3)	C21—C22—C23—C24	0.4 (5)
C9—C10—C11—C12	−1.3 (6)	C22—C23—C24—C19	−0.7 (5)
C10—C11—C12—O1	179.2 (3)	C20—C19—C24—C23	0.4 (5)
C10—C11—C12—C13	−0.4 (6)	C18—C19—C24—C23	179.6 (3)

### Hydrogen-bond geometry (Å, °)

**Table:**

*D*—H···*A*	*D*—H	H···*A*	*D*···*A*	*D*—H···*A*
O2—H2···N1^i^	0.82	1.98	2.762 (4)	159
O1—H1···N2^ii^	0.82	2.00	2.780 (4)	159

Symmetry codes: (i) *x*, *y*, *z*+1; (ii) *x*, *y*, *z*−1.
